# Splanchnic vein thrombosis in myeloproliferative neoplasms: risk factors for recurrences in a cohort of 181 patients

**DOI:** 10.1038/bcj.2016.103

**Published:** 2016-11-04

**Authors:** V De Stefano, A M Vannucchi, M Ruggeri, F Cervantes, A Alvarez-Larrán, A Iurlo, M L Randi, L Pieri, E Rossi, P Guglielmelli, S Betti, E Elli, M C Finazzi, G Finazzi, E Zetterberg, N Vianelli, G Gaidano, I Nichele, D Cattaneo, M Palova, M H Ellis, E Cacciola, A Tieghi, J C Hernandez-Boluda, E Pungolino, G Specchia, D Rapezzi, A Forcina, C Musolino, A Carobbio, M Griesshammer, T Barbui

**Affiliations:** 1 Institute of Hematology, Catholic University, Roma, Italy; 2 Center for Research and Innovation of Myeloproliferative Neoplasms, A.O.U. Careggi, University of Florence, Florence, Italy; 3 Ospedale San Bortolo, Vicenza, Italy; 4 Hospital Clínic IDIBAPS, Barcelona, Spain; 5 Department of Hematology, Hospital del Mar-IMIM, Barcelona, Spain; 6 Oncohematology Division, Fondazione IRCCS Ca' Granda, Ospedale Maggiore Policlinico, Milano, Italy; 7 Clinica Medica 1, Università di Padova, Padova, Italy; 8 Divisione di Ematologia, Ospedale San Gerardo, ASST Monza, Italy; 9 Hematology Division, ASST Papa Giovanni XXIII, Bergamo, Italy; 10 Skane University Hospital, Lund, Sweden; 11 Institute of Hematology and Medical Oncology, S. Orsola-Malpighi Hospital, University of Bologna, Bologna, Italy; 12 Department of Translational Medicine, Università del Piemonte Orientale, Vercelli, Italy; 13 University Hospital of Olomouc, Olomouc, Czech Republic; 14 Department of Hematology, Institute Meir Medical Center, Kfar Saba, Israel; 15 Haemostasis Unit, Department of Medical, Surgical and Advanced Technologies Sciences ‘G.F. Ingrassia', University of Catania, Catania, Italy; 16 Arcispedale Santa Maria Nuova—IRCCS, Reggio Emilia, Italy; 17 Hospital Clinico, Valencia, Spain; 18 A.O. Ospedale Niguarda Ca' Granda, Milano, Italy; 19 A.O. Universitaria, Policlinico di Bari, Italy; 20 A.O. Santa Croce e Carle, Cuneo, Italy; 21 IRCCS Ospedale San Raffaele, Milano, Italy; 22 A.O. Universitaria, Messina, Italy; 23 FROM Research Foundation, ASST Papa Giovanni XXIII, Bergamo, Italy; 24 Johannes Wesling Medical Center Minden, University of Bochum, Minden, Germany

## Abstract

We retrospectively studied 181 patients with polycythaemia vera (*n*=67), essential thrombocythaemia (*n*=67) or primary myelofibrosis (*n*=47), who presented a first episode of splanchnic vein thrombosis (SVT). Budd–Chiari syndrome (BCS) and portal vein thrombosis were diagnosed in 31 (17.1%) and 109 (60.3%) patients, respectively; isolated thrombosis of the mesenteric or splenic veins was detected in 18 and 23 cases, respectively. After this index event, the patients were followed for 735 patient years (pt-years) and experienced 31 recurrences corresponding to an incidence rate of 4.2 per 100 pt-years. Factors associated with a significantly higher risk of recurrence were BCS (hazard ratio (HR): 3.03), history of previous thrombosis (HR: 3.62), splenomegaly (HR: 2.66) and leukocytosis (HR: 2.8). Vitamin K-antagonists (VKA) were prescribed in 85% of patients and the recurrence rate was 3.9 per 100 pt-years, whereas in the small fraction (15%) not receiving VKA more recurrences (7.2 per 100 pt-years) were reported. Intracranial and extracranial major bleeding was recorded mainly in patients on VKA and the corresponding rate was 2.0 per 100 pt-years. In conclusion, despite anticoagulation treatment, the recurrence rate after SVT in myeloproliferative neoplasms is high and suggests the exploration of new avenues of secondary prophylaxis with new antithrombotic drugs and JAK-2 inhibitors.

## Introduction

Splanchnic vein thrombosis (SVT) encompasses hepatic veins (Budd–Chiari syndrome, BCS) and portal, mesenteric and splenic veins, and is a rare event in the general population: the annual incidence accounts for 0.8 per million for BCS, 0.7 per 100 000 for portal vein thrombosis and 2.7 per 100 000 for mesenteric vein thrombosis.^1, 2, 3, 4^ In these cases, the JAK2V617F mutation, the hallmark of myeloproliferative neoplasms (MPNs), can be detected in 13.6–26.6% of non-cirrhotic and non-malignant SVT patients, even in the absence of a clear diagnostic picture of MPN.^5^ In fully expressed Philadelphia-negative MPN, including polycythaemia vera (PV), essential thrombocythaemia and primary myelofibrosis, the more frequent sites of thrombosis are arterial vessels or leg veins and pulmonary embolisms (PEs) but also include unusual sites such as cerebral veins^6^ and SVT. These latter occur at much higher frequency (1–10%)^7^ than in the general population, where the incidence of these rare events is no more than 2–3 per 100 000.^1, 2, 3, 4^

Whether the recurrent events have a higher frequency than in the non-MPN population has not yet been assessed in a large series of patients with a well-established diagnosis of fully expressed MPN. To prevent recurrence, there is a general consensus that vitamin K-antagonist (VKA) should be prescribed long-term, very often in combination with cytoreductive therapy.^8, 9^ However, this recommendation is based on a consensus among the experts and is not universally accepted. A recent survey involving expert haematologists revealed a marked heterogeneity of choices on the duration of VKA and on the use of cytoreduction; moreover, this survey showed great uncertainty regarding the use of aspirin either alone or in association with VKA.^10^

In this study, we retrospectively collected a cohort of 181 SVT index events occurring in strictly defined patients with PV, essential thrombocythaemia and primary myelofibrosis managed in the clinical practice of 23 haematologic European centres. We describe here the pattern of presentation and the rate and risk factors of recurrent thrombotic events, and report the bleeding associated with secondary prophylaxis therapy.

## Patients and methods

### Study patients

A retrospective study was conducted across 23 centres within the European Leukaemia Network on patients with a diagnosis of MPN according to the World Health Organization 2008 criteria,^11^ after the approval of the ethics committees (primary approval by the central ethics committees of the coordinating centre was obtained on 2 October 2014).

The participating centres were asked to select from their consecutive patients with MPN, the ones who had had venous thromboembolism (VTE) objectively documented from January 2005, including deep venous thrombosis (DVT) of the limbs, PE, thrombosis of the cerebral and splanchnic veins (hepatic, portal, mesenteric and splenic veins) and thrombosis of the retinal vein, and who had received a course of VKA or direct oral anticoagulants. The data were collected in an electronic system. Each centre reported the total number of medical files by data input into an electronic database developed to record all study data; patients were de-identified with an alphanumeric code to protect personal privacy.

The details of the survey procedure and the results obtained in the patients with DVT of the legs and/or PE have been previously published.^12^ Briefly, for each patient, the following information was recorded: demographic data, WHO diagnosis, location of thrombosis, method of objective diagnosis, the presence of microvascular disturbances (that is, erythromelalgia, transient ocular attacks, pulsatile headache, dizziness and tinnitus) or constitutional symptoms (that is, pruritus, fatigue, night sweats, fever, weight loss and pain in the limbs), mutational profile, results of the laboratory investigation for thrombophilia, full blood count at diagnosis and at thrombosis, and the presence of cardiovascular risk factors (that is, history of previous thrombosis before the index event, smoking habit, hypertension, dyslipidaemia and diabetes). Moreover, the presence of circumstantial risk factors at the time of any episode of VTE such as surgery, pregnancy, puerperium (until 6 weeks from delivery), oral contraceptive intake, hormone replacement therapy, trauma, leg cast, prolonged bed immobilization (>10 days) and long travel (>8 h) was also recorded; in the absence of the previously mentioned risk factors, VTE was considered unprovoked. Finally, the data regarding cytoreductive or antithrombotic treatment after VTE, the duration of the treatment and the reasons for discontinuation of the treatment was recorded. A diagnosis of VTE was accepted only if it was confirmed by objective methods according to current clinical practice, as previously reported,^12^ and was defined as a positive result using techniques such as angiography, ultrasonography, computerized tomography or nuclear magnetic resonance.

### Outcomes

The aim of this study was to determine the rate of recurrent thrombosis in patients recruited in the general database, who had an SVT as an index event. SVT was defined as thrombosis of the hepatic, portal, mesenteric and splenic veins.

Venous or arterial thrombotic events that occurred after the index event were recorded only if objectively documented. Recurrent SVT was defined as an objectively established thrombus extension or occurrence in a previously patent segment, as previously defined.^13^ The following new manifestations, when objectively proven, were also defined as recurrent VTE: DVT of the arms or the limbs, PE and occlusion of cerebral veins. Finally, new arterial thrombotic events included ischaemic stroke, transient ischaemic attack, acute myocardial infarction, unstable angina pectoris and peripheral arterial thrombosis, when objectively diagnosed, as previously reported.^12, 13, 14^

Major bleeding was defined according to the criteria of the International Society on Thrombosis and Haemostasis^15^ when it was fatal and/or was symptomatic in a critical area or organ, such as intracranial bleeding, intraspinal bleeding, intraocular bleeding, retroperitoneal bleeding, intra-articular bleeding, pericardial bleeding, intramuscular bleeding with compartment syndrome and/or bleeding that led to a reduction of 2 g/dl or more in the haemoglobin concentration and/or necessitated the transfusion of two or more blood units.

### Statistical methods

For continuous variables, the median and the range are provided. The annual incidence of recurrent thrombosis was calculated by dividing the number of events by the total number of patient years (pt-years). Differences in the proportions were estimated using the Fisher's exact test (statistical significance threshold set at *P*<0.05).

A multivariable model, including age >60 years, thrombosis history, cardiovascular risk factors, Hb >15 g/dl, Hct >45%, white blood cell (WBC) count >14 × 10^9^/l, platelet count >500 × 10^9^/l, splenomegaly, unprovoked event, BCS as index event versus other SVT index events, VKA treatment and other treatments, was performed to identify the significant predictors of recurrence or bleeding. Starting with all candidate variables, backward selection was used to test whether the deletion or retention of each variable improved the model, repeating this process until no further improvement was possible.

The probability of recurrence as a function of time was estimated using the Kaplan–Meier method by analysing the interval between the index thrombosis and a recurrent thrombotic event (uncensored observations) or the duration of time until death, or the time elapsed until the patient's final visit to the centre (censored observations). The thrombosis recurrence-free survival was compared between the groups using the log-rank test (statistical significance threshold set at *P*<0.05) and the relative risk of recurrence was estimated as a hazard ratio (HR) with a 95% confidence interval using a Cox proportional hazard regression model.

## Results

### Clinical and laboratory features of the patient cohort

Overall, 181 patients with SVT at MPN diagnosis (58%) or during the course of the disease were recruited (Table 1). The female sex was prevalent (65.2%); at the time of the index event, only a minority of patients (22.1%) were over 60 years of age and 28.2% were under 40 years of age.

BCS and portal vein thrombosis were observed in 31 (17.1%) and 109 (60.3%) patients, respectively. The remaining 41 patients presented with isolated thrombosis of the mesenteric or splenic veins. The great majority were splenomegalic (median 4 cm below the costal margin) and one quarter reported microvascular disturbances and constitutional symptoms. Almost all (93.3%) carried the JAK2V617F mutation and 35% tested positive for genetic or acquired thrombophilia. No risk circumstance provoking SVT was present in the majority of the patients (85.6%). Of note, 113 patients (62.4%) had elevated blood counts, defined as Hct >45% and/or WBC>14 × 10^9^/l and/or platelet count >500 × 10^9^/l; no patient had WBC and platelet counts lower than 3.0 × 10^9^ and 100 × 10^9^/l, respectively.

### Incidence of new vascular events after the index event and risk factors

All patients received a course of low-molecular-weight heparin as initial therapy, followed in the majority of the patients by VKA (153/181, 84.5%) as secondary prophylaxis (Table 2). Among patients on VKA, the drug was continued indefinitely in 136 (88.9%) for a median follow-up time of 3.5 years (range 8 months–15.8 years) and was discontinued in 17 (9.3%) after a median time of 2 years (range 1 month–6.2 years). The reasons for stopping VKA included a pre-established plan by the centres in six cases and major bleeding in three; in the remaining patients, VKA was discontinued because of the patient's preference or bleeding risk judged to be excessive by the care physician (for example, onset of thrombocytopenia during the course of the disease).

The overall observation time recorded after the SVT index event was 735 years (median 3.2, range 1 month to 15.8 years). There were 31 new episodes of thrombosis in 31 patients, with an overall incidence rate (IR) of 4.2 per 100 pt-years (95% confidence interval (95% CI): 2.9–5.9); 17 thrombotic events (54.8%) occurred within 2 years from the date of the index SVT. Sites of events were in the splanchnic veins in 45% and at other sites in 55%: cerebral veins, venous legs with or without PE and arterial occlusions (Table 3).

No significant differences were found between the patients who had a recurrence and those who did not regarding diagnosis of PV (*P*=0.15), essential thrombocythaemia (*P*=0.41) and MF (*P*=0.82), the presence of JAK2V617F mutation (*P*=0.69) and thrombophilia (*P*=0.13).

**Figure 1:**
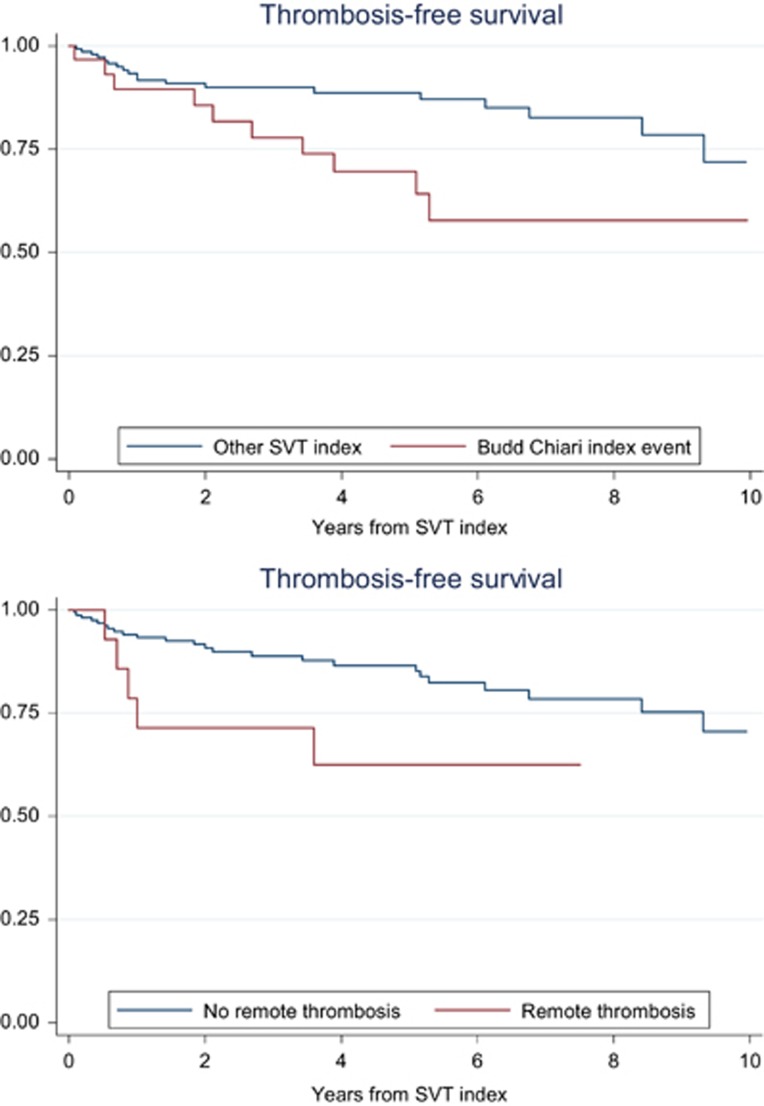
Thrombosis free-survival in patients with SVT index event according to the type of first event (BCS; HR: 2.38, 95% CI: 1.08–5.38, *P*=0.02; upper panel) and history of remote thrombosis (HR: 2.57, 95% CI: 0.99–6.88, *P*=0.04; lower panel).

**Figure 2:**
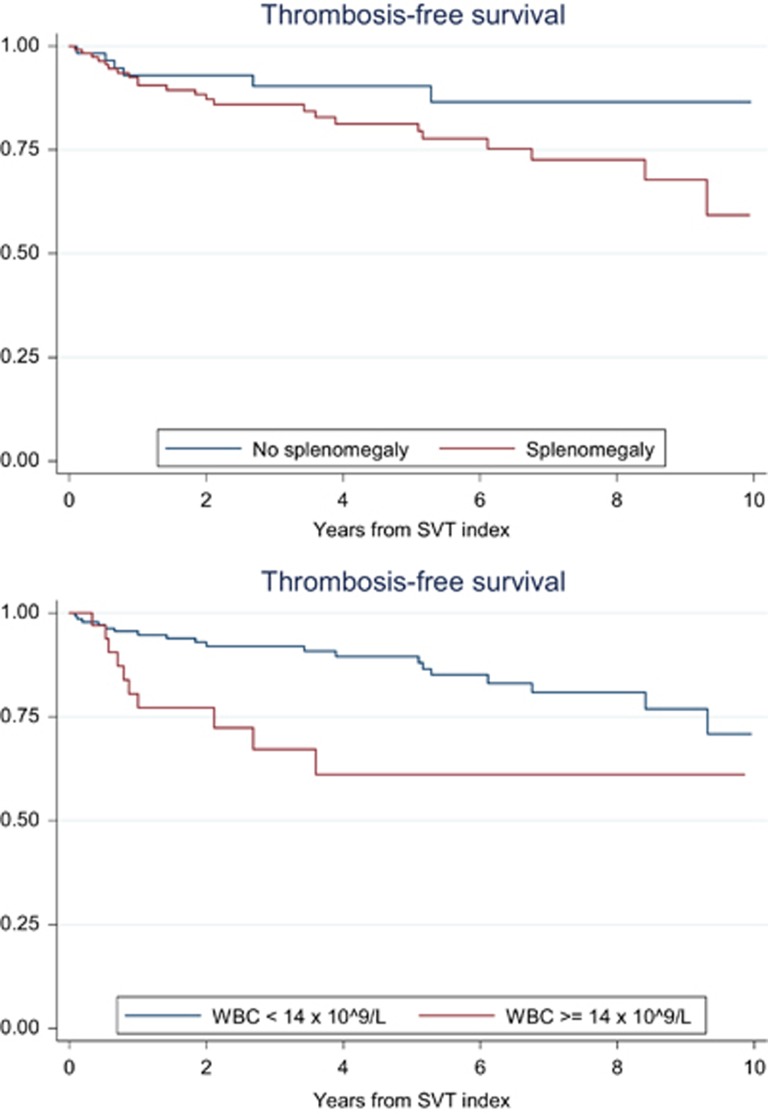
Thrombosis free-survival in patients with SVT index event according to presence of splenomegaly (HR: 2.24, 95% CI: 1.00–6.71, *P*=0.05; upper panel) and the presence of leukocytosis at the diagnosis (HR: 2.81, 95% CI: 1.17–6.34, *P*=0.08; lower panel).

Backward logistic regression analysis showed an independent association with the new thrombotic event for BCS (HR: 3.03, 95% CI: 1.37–6.69), history of previous thrombosis (HR: 3.62, 95% CI: 1.22–10.78), splenomegaly (HR: 2.66, 95% CI: 1.06–6.64) and leukocytosis higher than the highest quartile, 14 × 10^9^/l (HR: 2.8, 95% CI: 1.32–6.28). In particular, patients with BCS had an incidence rate of new events of 8.0 per 100 pt-years (95% CI: 4.0–14.4) that was significantly higher than in those with thrombosis of portal or other abdominal sites (Table 4). This difference was due to an increased rate of venous events in BCS patients, whereas no difference between the two groups was noticed in the rate of new arterial thromboses; of note, in patients with BCS there was a threefold increase in risk of recurrent SVT in respect to that of patients with other index SVT (5/31, 16.1% versus 9/150, 6%, odds ratio: 3.01, 95% CI: 0.93–9.71, *P*=0.06). The time to new events according to the presence of such risk factors is shown in the Kaplan–Meier curves of Figures 1 and 2.

### Effect of VKA antithrombotic treatment on the incidence of recurrent thrombosis

Patients on VKA (*n*=136) experienced 23 new thrombotic events during a follow-up of 585 years, corresponding to a rate of 3.9 (95% CI: 2.4–5.8) per 100 pt-years. Fourteen events (60.8%) involved venous vesselsnine recurrent SVT, two DVT of the legs, two PE and one cerebral vein thrombosis. The remaining events were ischaemic stroke (*n*=3), acute coronary syndrome (*n*=2), peripheral artery thrombosis (*n*=2) and retinal artery thrombosis (*n*=1); the site of one event was not specified. In 13 cases, the international normalized ratio value at the time of recurrence was available, being within the therapeutic range 2.0–3.0 in six cases (range 2.10–2.80), <2.0 in five cases (range 1.70–1.85) and >3.0 in two cases (range 5.03–7.10).

Patients who discontinued VKA (*n*=17) and patients who never received VKA or direct oral anticoagulant prophylaxis (*n*=25) were followed up for 146 years and developed 4 and 4 events, respectively, corresponding to an overall annual rate of 5.4 (95% CI: 2.3–10.7) per 100 pt-years, which was not significantly different from that observed in patients receiving VKA (*P*=0.41). Five events (62.5%) were recurrent SVT. The remaining events were myocardial infarction (*n*=1) and peripheral artery thrombosis (*n*=1); the site of one event was not specified.

The comparison between the patients with ongoing VKA and those off VKA at the time of recruitment in the survey was repeated by analysis on-treatment. Among the patients who received VKA after the index event for a limited period of time, the total observation time was 36 pt-years on VKA; therefore, the overall pt-years on VKA and off VKA were 621 and 110, respectively. Accordingly, the rate of recurrences per 100 pt-years with or without VKA was 3.7 (95% CI: 2.3–5.5) and 7.2 (95% CI: 3.1–14.3), respectively (*P*=0.09). The 4 pt-years attributed to the three patients on direct oral anticoagulants were not computed in the analysis; none of these latter patients had a recurrent thrombosis.

The rate of new thrombotic events in patients receiving antiplatelet agents alone or in association with VKA (*n*=16) was 4.3 (95% CI: 0.5–15.7) per 100 pt-years and the combination of aspirin with VKA did not produce any advantage on the overall rate of thrombosis (*P*=0.79). However, all the arterial thrombotic events occurred in the absence of antiplatelet agents.

### Effect of cytoreductive treatment on the incidence of recurrent thrombosis

Cytoreduction (mostly hydroxyurea) was administered to 130 patients (71.8% of the cohort) and was combined with VKA in 107 (82.3%). Patients with or without cytoreduction did not differ in the rate of PV, essential thrombocythaemia or MF diagnosis, age >60 years, BCS as index event, Hb >15 g/dl, WBC count >14 × 10^9^/l, platelet count >500 × 10^9^/l, splenomegaly and VKA treatment (data not shown). In patients receiving cytoreduction 23 recurrent events were recorded over 537 pt-years (IR 4.2 per 100 pt-years, 95% CI: 2.7–6.4) and in patients without cytoreductive treatment 8 recurrent events were recorded over 198 pt-years (IR 4.0 per 100 pt-years, 95% CI: 1.7–7.9; *P*=0.94). The analysis of the patients receiving both VKA and cytoreduction disclosed 20 recurrences over 471 pt-years (IR 4.2 per 100 pt-years, 95% CI: 2.5–6.5). Finally, four patients received ruxolitinib and had no recurrence after the SVT index event.

Overall, in patients not receiving cytoreduction after the index event, Hb >15 g/dl and/or WBC count >14 × 10^9^/l, and/or a platelet count >500 × 10^9^/l was recorded at the time of the recurrent thrombosis in three of eight cases with recurrences (37.5%). Similar blood values indicating a poor control of cell proliferation were observed at the time of the recurrence in 14 of the remaining 23 patients (60.8%) who had recurrent thrombosis during cytoreduction.

### Incidence rate of major bleeding

Major bleeding occurred in 16 patients, with a rate of 2.1 (95% CI: 1.3–3.5) per 100 pt-years and was intracranial in 4 cases (Table 3). Thirteen events (81.2%) occurred in patients on VKA, one of which was a fatal intracranial haemorrhage. Two bleeding events occurred during treatment with ticlopidine; the remaining patient was not receiving any antithrombotic treatment. The incidences of major bleeding in patients taking and not taking VKA were 2.0 (95% CI: 1.1–3.5) and 2.7 (95% CI: 0.5–7.9) per 100 pt-years, respectively (*P*=0.67; on-treatment analysis).

A tendency towards a significantly higher IR of major bleeding was recorded in patients receiving cytoreduction (2.7 per 100 pt-years, 95% CI: 1.5–4.6) in respect to those without cytoreductive treatment (0.5 per 100 pt-years, 95% CI: 0.0–2.8; *P*=0.06).

### Development of haematological transformation and solid cancer

During the follow-up (median 3.2 years), the progression to overt myelofibrosis and acute myeloid leukaemia was ascertained in 11 (6.1%) and 4 patients (2.2%), respectively; solid cancer was recorded in 4 patients (2.2% Table 3). The rate of haematological transformation tended to be higher in patients who had SVT during the follow-up after MPN diagnosis in comparison with patients who had SVT at the MPN diagnosis (11.8% vs 5.7%, *P*=0.15).

The disease transformation was unrelated to recurrent thrombotic events (odds ratio for transformation 1.8, 95% CI: 0.5–6.3, *P*=0.29).

## Discussion

Despite the large number of reports of retrospective or prospective case series and meta-analyses concerning the association between SVT and MPN,^5^ the studies on prophylaxis after MPN-related SVT are limited to small series of no more than 50 patients.^13, 16, 17, 18, 19, 20, 21^ Our survey is the largest study cohort of MPN-related SVT ever reported, describing in detail the treatments after the index event, the subsequent outcomes and the risk factors for recurrence.

SVT was the heralding manifestation of MPN in 58% of the patients and followed the diagnosis of MPN in 42% this result is substantially aligned with the results of a meta-analysis in which 36% of the MPN-related SVT occurred during the follow-up after diagnosis.^22^ The most frequent site of thrombosis was the portal-mesenteric axis and BCS accounted for 17% this latter frequency is slightly higher than the value observed in the general population, where BCS accounts for 5 to 12% of SVT.^13, 23, 24^ The majority of patients were young females (65%), with a median age of 48 years, in agreement with previous MPN series^16, 19, 25^ and contrasting with the gender and older median age observed in the general population.^13, 23^

Inherited or acquired thrombophilia was found in one-third of the evaluated patients, with no difference compared with the diagnostic yield obtained in the general patient series.^1, 13, 23^ Of note, almost all patients (93%) carried the JAK2 V617F mutation, confirming the strength of association between this mutation and vascular events.^5, 26^

After a median follow-up of 3.2 years, 31 new thromboses were recorded, including both splanchnic and other venous, as well as arterial occlusions. The overall incidence rate of recurrent thrombosis was 4.2 per 100 pt-years, which is consistent with the prospective incidence of 5.9 per 100 pt-years reported in a small series of MPN-related SVT.^13^

Multivariable analysis revealed that BCS, history of thrombosis, splenomegaly and leukocytosis were independently associated with recurrence. The high incidence of recurrences in BCS was also reported by others, both in MPN patients^20, 21^ and in the non-MPN population.^23^ A predictive role for incident thrombosis in prior thrombotic history^8, 9^ and leukocytosis^14, 27, 28^ has been consistently found in MPN patients, including both arterial and venous thrombosis.

Interestingly, 38% of the patients had a blood cell count without hypercythaemia, which is a finding quite common in MPN-related SVT;^25^ moreover, no relevant leukopenia or thrombocytopenia was recorded. Therefore, anticoagulation was considered safe and was administered to 95% of patients, only a fraction of whom (71%) received cytoreductive therapy; this practice was likely to be due to the reluctance to use cytoreductive drugs in cases without overt elevated blood counts. However, cytoreductive treatment did not reduce the incidence rate of recurrent thrombotic events; nevertheless, it should be highlighted that 17 of the 31 events (55%) occurred in patients with hypercythaemia not receiving cytoreduction or in patients who failed to reach the haematological response in spite of cytoreduction.

Finally, splenomegaly emerged as an independent risk factor for recurrent thrombosis in our series with SVT. This result is consistent with other observations reporting a role of splenomegaly for new episodes of thrombosis in patients without SVT as well,^29, 30^ even though this finding has not been confirmed by other researchers.^31^ It has been speculated that splenomegaly should be considered an index of more intensive myeloproliferation; however, its association with recurrent SVT may also be due to a more extensive occlusion of the hepatic-portal venous axis. In patients with SVT, the JAK2/JAK1 inhibitor Ruxolitinib has shown significant activity in reducing the spleen volume,^32^ but no data are available thus far on whether this finding is associated with fewer recurrences. However, as reported in a recent meta-analysis, there is evidence suggesting that the drug may reduce the rate of total major thrombosis in myelofibrosis and PV.^33^ In our series only four patients received ruxolitinib, so that no conclusion can be drawn on this issue.

The incidence rate of recurrence in our patients was 3.9 per 100 pt-years in patients on VKA and 7.2 per 100 pt-years in patients off VKA. This frequency is a new finding and agrees with other studies of SVT in which the estimates were performed in the general population with SVT, including only a minority of MPN. In these reports, the frequency of recurrence ranged from 1.3 to 5.6 per 100 pt-years on VKA and from 3.3 to 11.9 per 100 pt-years after VKA discontinuation.^13, 34, 35, 36^ Notably, in patients with non-splanchnic DVT on long-term treatment with VKA, the recurrence rate is expected to be below 1 per 100 pt-years.^37^ The 2.0% rate of major bleeding on VKA among our cases was similar to the rate in SVT without MPN (from 1.2 to 3.9% pt-years)^13, 34, 35, 36^ and was slightly higher than expected in patients with non-splanchnic DVT on long-term VKA treatment (0.9 per 100 pt-years).^37^ These indirect comparisons suggest that in SVT, with or without MPN, antithrombotic prophylaxis is associated with an inferior efficacy and safety profile compared with other DVT cases. Therefore, antithrombotic prophylaxis in SVT, particularly in BCS, is an unmet clinical need and warrants prospective studies exploring the role of new direct oral anticoagulants^38^ and the new JAK2 inhibitors in MPN patients.

## Figures and Tables

**Table 1:** Clinical features of the cohort at the time of the index event

Male/female, *N* (%)	63/118 (34.8/65.2)
Age, years, median (range)	48 (29–74)
<40 years, *n* (%)	51 (28.2)
40–60 years, *n* (%)	90 (49.7)
⩾60 years, *n* (%)	40 (22.1)
	
*Diagnosis*, N *(%)*	
PV	67 (37.0)
ET	67 (37.0)
PMF	47 (26.0)
Years from diagnosis to index thrombosis, mean (±s.d.)	2.01 (3.87)
Diagnosis at the time of index thrombosis, *n* (%)	105 (58)
	
Hb gr/dl, median (range)	13.4 (6.3–24.0)
Hct %, median (range)	42.1 (22.0–70.0)
WBC count × 10^9^/l, median (range)	9.3 (3.0–50.0)
Platelet count × 10^9^/l, median (range)	424 (100–3000)
	
JAK2 mutation, *N*/*N* tested (%)	166/178 (93.3)
CALR mutations, *N*/*N* tested	4/25 (16.0)
MPL mutations, *N*/*N* tested	1/28 (3.6)
Exon 12 mutations, *N*/*N* tested	0/14 (0.0)
	
Abnormal karyotype, *N*/*N* tested (%)	8/65 (12.3)
Microvascular disturbances, *N* (%)	19 (10.5)
Constitutional symptoms, *N* (%)	28 (15.5)
Palpable splenomegaly, *N* (%)	119 (65.8)
Cm below costal margin, median (range)	4 (1–25)
	
*Localization of index thrombosis*, N *(%)*	
Hepatic vein thrombosis (BCS)	31 (17.1)
Portal vein thrombosis	109 (60.3)
Mesenteric vein thrombosis	18 (9.9)
Splenic vein thrombosis	23 (12.7)
	
Unprovoked thrombosis, *N* (%)	155 (85.6)
Provoked by, *n* (%)	26 (14.4)
Oral contraceptives	8 (4.4)
Hormone replacement therapy	3 (1.7)
Infection	3 (1.7)
Pregnancy/puerperium	1 (0.6)
Cancer	1 (0.6)
Surgery	7 (3.9)
Liver disease	3 (1.7)
	
*Risk factors for index thrombosis*	
History of thrombosis, *N* (%)	14 (7.7)
History of arterial thrombosis, *n* (%)	5 (2.8)
History of venous thrombosis, *n* (%)	8 (4.4)
Presence of at least one vascular risk factor, *N* (%)	52 (28.7)
Smoking habit	19 (10.5)
Hypertension	28 (15.5)
Dislipidemia	7 (3.4)
Diabetes	5 (2.8)
Presence of thrombophilia*, *N*/*N* tested (%)	42/120 (35.0)
Inherited thrombophilia, *N*/*N* tested (%)	21/120 (17.5)

Abbreviations: BCS, Budd Chiari syndrome; ET, essential thrombocythaemia; Hb, haemoglobin; PMF, primary myelofibrosis; PV, polycythaemia vera; WBC, white blood cell.

Deficiency of antitrombin (*n*=1), deficiency of protein C or protein S (*n*=7), factor V Leiden and/or prothrombin G20210A (*n*=13), increased levels of homocysteine (*n*=16), antiphospholipids (*n*=6).

**Table 2:** Treatment after the index thrombosis

	*Total (%)*	*Cytoreduction*^a^	
		*Yes*	*No*
VKA	143 (79.0)	101 (70.6)	42 (29.4)
VKA+antiplatelets	10 (5.5)	6 (60.0)	4 (40.0)
Antiplatelets	6 (3.3)	6 (100)	2 (0)
Heparin	10 (5.5)	7 (70.0)	3 (30.0)
DOACs	3 (1.7)	2 (66.7)	1 (33.3)
No antithrombotic treatment	9 (5.0)	8 (88.9)	1 (11.1)
Total (%)	181 (100)	130 (71.8)	51 (28.2)

Abbreviations: DOAC, direct oral anticoagulant; VKA, vitamin K-antagonists.

a Hydroxyurea in 108/130 cases (83%).

**Table 3:** Overall incidence of major outcomes after the index SVT

	*Events,* n *(%)*	*Incidence rate per 100 pt-years (95% CI)*
Thrombotic events	31 (17.1)	4.2 (2.9–5.9)
*Venous thrombosis*	19 (10.5)	2.5 (1.6–4.0)
Recurrent SVT		1.9 (1.1–3.1)
Hepatic vein thrombosis	3 (1.7)	
Portal vein thrombosis	4 (2.2)	
Mesenteric vein thrombosis	6 (3.1)	
Splenic vein thrombosis	1 (0.6)	
Venous thrombosis at other sites		0.6 (0.2–1.5)
DVT	2 (1.1)	
PE	2 (1.1)	
Cerebral vein thrombosis	1 (0.6)	
*Arterial thrombosis*	10 (5.5)	1.3 (0.7–2.4)
Unstable angina	1 (0.6)	
Myocardial infarction	2 (1.1)	
Ischaemic stroke	3 (1.7)	
Peripheral artery thrombosis	3 (1.7)	
Retinal artery thrombosis	1 (0.6)	
Not specified	2 (1.1)	
		
Major bleeding	16 (8.8)	2.1 (1.3–3.5)
CNS	4 (2.2)	
Gastrointestinal	6 (3.1)	
Muscle hematoma	2 (1.1)	
Unspecified	4 (2.2)	
		
Hematologic evolutions	15 (8.3)	2.0 (1.2–3.3)
MF	11 (6.1)	
AML	4 (2.2)	
		
Solid cancer	4 (2.2)	0.5 (0.2–1.3)
Brain glioblastoma	1 (0.5)	
Breast cancer	1 (0.5)	
Nose skin cancer	1 (0.5	
Pancreas cancer	1 (0.5)	
		
Deaths	14 (7.7)	1.9 (1.1–3.1)
ICH	1 (0.6)	
Infection	1 (0.6)	
MF	1 (0.6)	
AML	4 (2.2)	
Solid tumour	2 (1.1)	
Other	2 (1.1)	
Unknown	3 (1.7)	

Abbreviations: AML, acute myeloid leukemia; CI, confidence interval; CNS, central nervous system; DVT, deep vein thrombosis; ICH, intracranial haemorrhage; MF, myelofibrosis; PE, pulmonary embolism; pt-years, patient years; SVT, splanchnic vein thrombosis

**Table 4:** Sub-analysis of major outcomes and type of SVT index event (BCS vs other SVT)

	*Index BCS (*n*=31) pt-years=136*	*Other index SVT (*n*=150) pt-years=599*	P*-value*
Thrombotic events, *n* (%)	11 (35.4)	20 (13.3)	0.006
Incidence rate, per 100 pt-years (95% CI)	8.0 (4.0–14.4)	3.3 (2.0–5.1)	0.01
Venous thrombosis, *n* (%)	7 (22.5)	12 (8.0)	0.02
Incidence rate, per 100 pt-years (95% CI)	5.1 (2.0–10.6)	2.0 (1.0–3.4)	0.03
Arterial thrombosis, *n* (%)	3 (9.6)	7 (4.6)	0.37
Incidence rate, per 100 pt-years (95% CI)	2.2 (0.4–6.4)	1.1 (0.4–2.4)	0.34
Major bleeding	3 (9.6)	13 (8.6	0.74
Incidence rate, per 100 pt-years (95% CI)	2.2 (0.4–6.4)	2.1 (1.1–3.7)	0.97
Deaths	2 (6.4)	12 (8.0)	1.00
Incidence rate, per 100 pt-years (95% CI)	1.4 (0.1–5.3)	2.0 (1.0–3.4)	0.64

Abbreviations: CI, confidence interval; BCS, Budd–Chiari syndrome; pt-years, patient years; SVT, splanchnic vein thrombosis.
